# Antiphospholipid Syndrome as an Under-Recognized Cause of Ischemic Stroke in Older Adults: A Retrospective Case Series

**DOI:** 10.7759/cureus.116600

**Published:** 2026-09-20

**Authors:** Seyed Mostafa Razavi, Mahika Khurana, Mariami Gabadadze, Balaganesh Natarajan, Poojitha Sushma Malla, Eman Elmasry Eldamarany Khalifa, Ahmed Abd Elazim

**Affiliations:** 1 Department of Neurology, University of South Dakota Sanford School of Medicine, Sioux Falls, USA; 2 Neurology, Maternity and Children Hospital, Hofuf, SAU

**Keywords:** anticoagulation, antiphospholipid syndrome, elderly, embolic stroke of undetermined source, ischemic stroke, lupus anticoagulant

## Abstract

Background, objective, and methods: Antiphospholipid syndrome (APS) is classically regarded as a cause of thrombosis and stroke in younger patients and is often considered a rare etiology beyond the sixth decade. We retrospectively describe seven older adults (ages 67-90 years; median 76; four females) with ischemic stroke in whom antiphospholipid antibodies (aPL) were identified during evaluation, most after one or more cerebrovascular events.

Results: Six of the seven had radiographic or clinical evidence of prior cerebrovascular disease, and several had experienced recurrent events despite antiplatelet or anticoagulant therapy. Antibody profiles were heterogeneous, ranging from triple positivity with confirmed persistence to isolated lupus anticoagulant and IgM-only positivity, and competing stroke mechanisms (patent foramen ovale, large-artery atherosclerosis, and small-vessel disease) were frequently present. All patients were transitioned to a vitamin K antagonist.

Conclusions: This series illustrates that aPL-associated stroke does occur in older adults and supports considering APS in the differential of cryptogenic or recurrent ischemic stroke in this population, while underscoring the need for high-specificity, persistently confirmed testing before committing a patient to lifelong anticoagulation.

## Introduction

Antiphospholipid syndrome (APS) is an acquired thrombophilia defined by vascular thrombosis or pregnancy morbidity in the persistent presence of antiphospholipid antibodies (aPL): lupus anticoagulant (LA), anticardiolipin (aCL), and anti-β2-glycoprotein I (aβ2GPI) antibodies [1,2]. In population-based data, the mean age at APS diagnosis is in the mid-fifties [3], with large cohort studies documenting substantial associated morbidity and mortality over long-term follow-up [4], and most teaching frames aPL testing as a priority in younger patients with stroke [5]; in adults older than 50 years, APS is often described as a very uncommon cause of ischemic stroke [5-7]. Yet aPL are also identified in a meaningful proportion of unselected patients with cerebrovascular events [8], and an observational cohort recently reported that more than a quarter of thrombotic APS patients had disease onset at age 60 or older, with stroke significantly more common as the presenting event in this group than in younger patients [9]. Recognizing APS in older adults is clinically consequential because it shifts secondary prevention from antiplatelet therapy toward anticoagulation. We report the case of seven older adults with ischemic stroke and antiphospholipid antibodies and use the series to examine both the case for considering APS in this population and the diagnostic caution that the heterogeneity of aPL findings demands. This series aims to characterize the clinical and aPL profiles of seven older adults with ischemic stroke and propose a graded approach to attributing stroke to APS in this population.

## Materials and methods

We retrospectively identified seven consecutive patients aged 65 years or older admitted to our vascular neurology stroke service between January 2025 and June 2026 for ischemic stroke who were found to have positive aPL testing during their evaluation. Inclusion criteria were age ≥65 years, admission to the stroke service for ischemic stroke, and positive aPL testing obtained during that evaluation. Patients were excluded if they had a diagnosis of APS or systemic lupus erythematosus (SLE) established prior to the index stroke, if the index event was hemorrhagic rather than ischemic, or if antibody testing or clinical records were incomplete or unavailable. Antiphospholipid antibody testing was ordered at the treating physician's discretion rather than as a fixed screening protocol, so this series does not represent a consecutive or denominator-defined sample of all ischemic stroke admissions aged ≥65 years during the study period. Clinical data, neuroimaging interpretations, and laboratory results were abstracted from the medical record.

Lupus anticoagulant was assessed by dilute Russell viper venom time (DRVVT) screening (reference range 30.0-45.0 sec, confirmation reference range 29.0-38.0 sec, confirmation ratio reference range 0.00-1.40; lupus anticoagulant activity indicated by a screen-confirm delta >8.0 sec) with confirmatory mixing/neutralization studies (Sanford Reference Laboratory, Sioux Falls, SD, USA). Anticardiolipin and anti-β2-glycoprotein I IgG and IgM were measured by BioRad multiplex flow immunoassay (BioRad Laboratories, CA, USA), with results reported as qualitative positive or negative interpretations per manufacturer-defined thresholds.

Antibody profiles and, where available, repeat testing were interpreted in the context of the 2006 revised Sapporo (Sydney) criteria and the 2023 American College of Rheumatology and the European Alliance of Associations for Rheumatology (ACR/EULAR) classification criteria [1,2]. Stroke mechanism was classified using TOAST (Trial of Org 10172 in Acute Stroke Treatment) criteria [10]. Given the small, retrospective, single-center design, competing stroke mechanisms (patent foramen ovale or intracardiac shunt, large-artery atherosclerosis, and small-vessel ischemic disease) and a heterogeneous burden of vascular comorbidities were recognized as potential confounders in attributing the index stroke to aPL positivity. These are detailed by the patient and addressed further in the discussion.

All identifying information was removed; patients were designated patient #1 through patient #7. No protected health information is reported. This study was reviewed by the Sanford Health Institutional Review Board (STUDY00004383), which issued a 'not human research' determination, and thus review and approval were not required.

## Results

The cohort comprised four women and three men aged 67 to 90 years (median 76). Six of the seven had prior strokes, chronic infarcts on imaging, or other prior thromboembolism, and the index event was frequently embolic-appearing or multifocal. Demographics, vascular risk factors, and stroke characteristics are summarized in Table 1. Antiphospholipid antibody profiles, classification status, and antithrombotic management are summarized in Table 2.

**Table 1:** Patient demographics, vascular risk factors, and stroke characteristics CAD: Coronary artery disease; MI: Myocardial infarction; PCI: Percutaneous coronary intervention; ICA: Internal carotid artery; DVT: Deep vein thrombosis; PCA: Posterior cerebral artery; MCA: Middle cerebral artery; ACA: Anterior cerebral artery; PFO: Patent foramen ovale; TTE: Transthoracic echocardiogram; TIA: Transient ischemic attack; COPD: Chronic obstructive pulmonary disorder; CKD: Chronic kidney disease

Patient no.	Age/sex	Vascular risk factors	Prior cerebrovascular events	Index infarct (territory)	Competing mechanism considered
1	76/F	Hypertension, type 2 diabetes, hyperlipidemia, hypothyroidism, heavy tobacco (~56 pack-years)	Recurrent transient ischemic attack (TIA) on clopidogrel ~14 months after index event	Left thalamus, occipital, medial parietal (left posterior cerebral artery (PCA))	Small intracardiac shunt on transthoracic echocardiogram (TTE)
2	80/M	Coronary artery disease (CAD) with prior myocardial infarction (MI)(percutaneous coronary intervention (PCI)), hyperlipidemia, prior deep vein thrombosis (DVT), left internal carotid artery (ICA) stenosis (stented), morbid obesity, prediabetes	Left occipital and parietal infarcts ~7 mo earlier	Right anterior cerebral artery (ACA) (A3) with scattered infarcts in multiple territories; thrombectomy	Large-artery atherosclerosis; carotid stent with mural thrombus
3	71/M	None documented (no prior medical history on file); low-density lipoprotein (LDL) 112 mg/dL	None documented (first known event)	Left posterior middle cerebral artery (MCA) (M2): temporal, parietal, occipital	Mild cervical atherosclerosis only
4	90/F	Hypertension, hyperlipidemia, prior TIA, hypothyroidism, heavy alcohol use	Multiple prior infarcts (left frontal/temporal, bilateral cerebellar)	Left frontal and right cerebellar	Large patent foramen ovale (PFO) with right-to-left shunt; severe small-vessel disease; intracranial atherosclerosis
5	67/F	Hypertension, migraine, prior colon adenocarcinoma, chronic kidney disease, hyperlipidemia	Chronic left cerebellar infarct	Dorsal left frontal (left MCA)	No PFO; no significant stenosis
6	67/F	Hypertension, hypothyroidism, prediabetes, obesity, esophageal dysphagia	Ischemic stroke seven years earlier	Left parietal cortex	PFO (later closed); moderate left ICA-terminus stenosis
7	86/M	Hypertension, dyslipidemia, CAD with prior MI/ischemic cardiomyopathy, type 2 diabetes, recurrent DVT, chronic obstructive pulmonary disease (COPD), chronic kidney disease (CKD) stage III, colon cancer, aortic/iliac aneurysm	Chronic infarcts on imaging; recurrent DVT	Left MCA (M2)	Diffuse atherosclerosis incl; ulcerated aortic-arch plaque; chronic nonocclusive DVT

**Table 2:** The aPL antibody profiles, persistence, classification status, and antithrombotic management '+' denotes a result above the laboratory reference cutoff. Persistence refers to documented repeat positivity at least 12 weeks apart. The 2006 Sapporo [1] and 2023 ACR/EULAR [2] classification status reflects the profile and persistence documented at the last follow-up. 'Provisional' indicates that confirmatory or repeat testing had not yet been completed at the time of this report; 'Not met' indicates that the antibody profile is structurally excluded from that domain (isolated IgM under the 2023 criteria) regardless of future confirmatory testing. aPL: Antiphospholipid; aCL: Anticardiolipin antibody; aβ2GPI: Anti-β2-glycoprotein I antibody; DRVVT: Dilute Russell viper venom time; INR: International normalized ratio; SLE: Systemic lupus erythematosus; ACR: American College of Rheumatology; EULAR: European Alliance of Associations for Rheumatology

Patient no.	Lupus anticoagulant (DRVVT)	aCL IgG/IgM	aβ2GPI IgG/IgM	aPL profile	Persistence ≥12 wk	2006 Sapporo criteria met	2023 ACR/EULAR criteria met	Antithrombotic regimen
1	Positive (DRVVT 82.2 s, ratio 1.64; confirm ratio 1.75)	+/+ (IgM >112)	+/+ (IgM >112)	Triple positive (lupus anticoagulant, aCL, aβ2GPI; IgG and IgM)	Confirmed (2 panels ~12+ wk apart)	Meets criteria	Meets criteria	Warfarin (INR 2–3) + aspirin 81 mg
2	Not reported on available panel	+/–	+/–	Double positive, IgG (aCL + aβ2GPI)	Single panel	Provisional (persistence unconfirmed)	Provisional (persistence unconfirmed)	Warfarin + ticagrelor (coronary stents)
3	Positive (delta 9.8 s; DRVVT 58.7 s; confirm ratio 1.67)	+/–	+/–	Triple positive (lupus anticoagulant, aCL IgG, aβ2GPI IgG)	Confirmatory panel recommended (pending); SLE workup	Provisional (persistence unconfirmed)	Provisional (persistence unconfirmed)	Warfarin (INR 2–3), aspirin initially
4	Negative (delta 3.4 s; DRVVT 37.1 s)	–/+	–/+	Double positive, IgM only	Uncertain (LA neg earlier; IgM + later)	Provisional (persistence unconfirmed)	Not met (isolated IgM excluded)	Warfarin (INR 2–3) + aspirin 81 mg
5	Positive (delta 24.8 s; DRVVT 50.0 s; confirm ratio 1.36)	–/–	–/–	Isolated lupus anticoagulant	Single panel	Provisional (persistence unconfirmed)	Provisional (persistence unconfirmed)	Warfarin (no antiplatelet)
6	Positive (delta 16.7 s; DRVVT 45.5 s)	–/–	–/–	Isolated lupus anticoagulant	Single panel	Provisional (persistence unconfirmed)	Provisional (persistence unconfirmed)	Warfarin + clopidogrel
7	Negative (delta 1.0 s; DRVVT 34.9 s)	–/+	–/+	Double positive, IgM only	Single (recent); not yet confirmed	Provisional (persistence unconfirmed)	Not met (isolated IgM excluded)	Warfarin (INR 2.0) + aspirin 81 mg

Selected case features

Patient #1 presented with a left PCA-territory infarct and was treated with clopidogrel. A transient confusional episode consistent with recurrent ischemia occurred while on antiplatelet therapy, and subsequent testing revealed triple positivity with markedly elevated IgM titers, confirmed on repeat testing approximately 12 weeks later. She was transitioned to warfarin with low-dose aspirin. Patient #2 had extensive atherosclerotic and coronary disease and experienced recurrent strokes, including a large ACA infarct requiring thrombectomy and scattered infarcts in multiple territories, despite prior anticoagulation, with double IgG positivity (aCL and aβ2GPI). Patient #3, a 71-year-old man with no documented medical history, presented with a single but extensive left posterior MCA infarct and triple positivity; a 12-week confirmatory panel and evaluation for SLE were recommended. Patients #4 and #7 (ages 90 and 86, respectively) had IgM-only double positivity against a background of severe small-vessel disease and diffuse atherosclerosis, respectively, illustrating the attribution difficulty discussed below. Patient #4 later developed gross hematuria during a supratherapeutic international normalized ratio (INR), a reminder of the bleeding hazard of anticoagulation in the very elderly. Patients #5 and #6 (both aged 67) had isolated lupus anticoagulant positivity and were managed with warfarin. The MRI images from patients #4, #6, and #7, revealing new and old strokes, are featured in Figure 1. 

**Figure 1:**
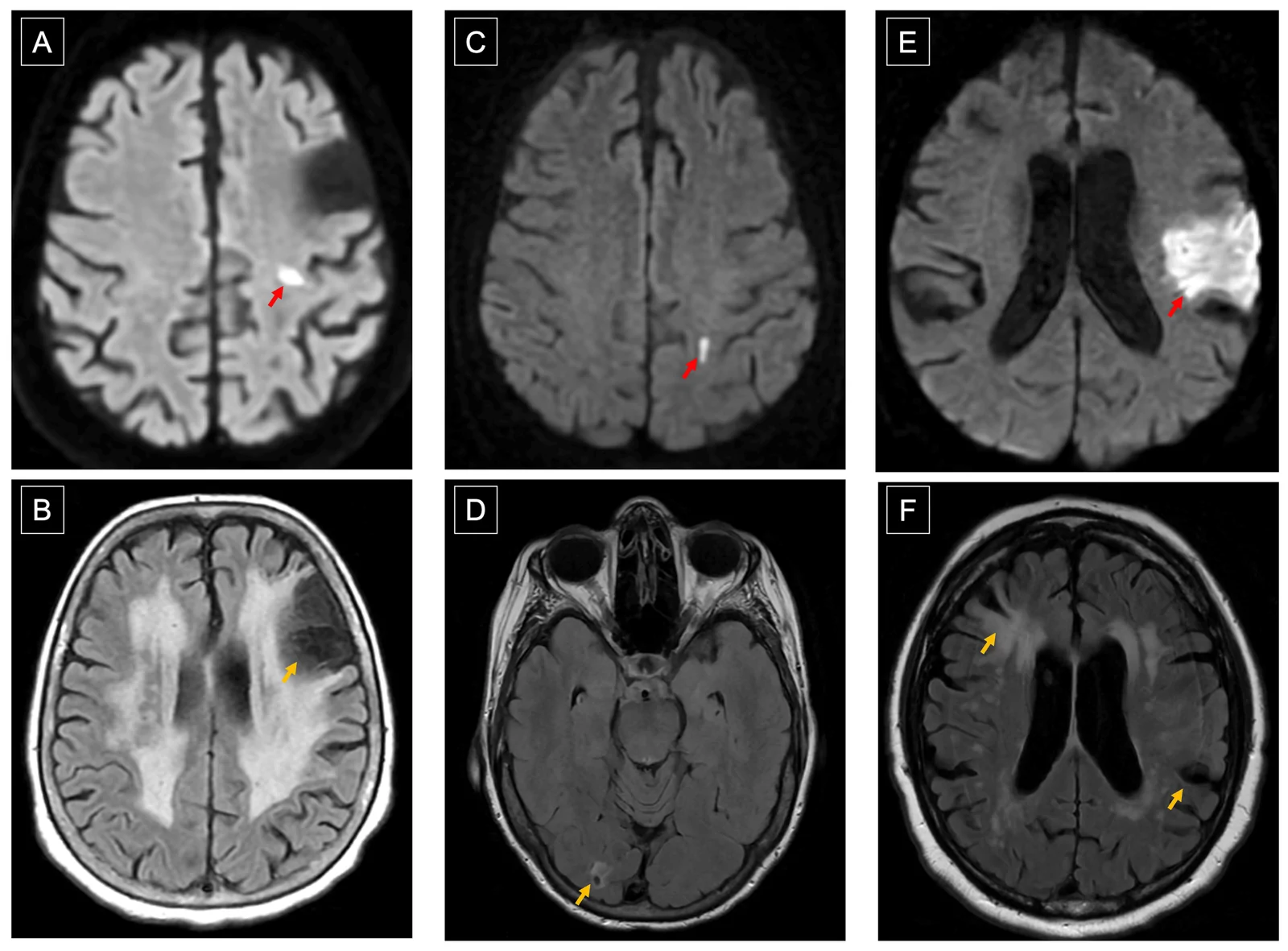
Diffusion-weighted images (panels A, C, and E) and fluid-attenuated inversion recovery (FLAIR) (panels B, D, and F) from the MRI of patients #4, #6, and #7 Red arrows: Acute infarct on diffusion-weighted image; Yellow arrows: Chronic infarcts on FLAIR, distinct from the diffuse small-vessel ischemic change that is also present Panels A and B (patient #4): Acute left frontal infarct (red arrow); Panels C and D (patient #6): Acute left parietal infarct (red arrow); Panels E and F (patient #7): Acute left MCA (M2)-territory infarct (red arrow). All images are original and were obtained during routine clinical care of the patients described in this series. FLAIR: Fluid-attenuated inversion recovery; MCA: Middle cerebral artery

## Discussion

These seven patients, with a median age of 76 years, reinforce a point that runs against common teaching: aPL antibodies are identifiable in older adults presenting with ischemic stroke, and in some cases APS may be a contributing factor. This is consistent with cohort data showing that elderly-onset APS does occur and more often presents with stroke than with venous thromboembolism compared with younger-onset APS, though age itself is a stroke risk factor and a causal relationship cannot be definitively established [9], with the observation that aPL are detectable in roughly one in five patients with acute cerebrovascular events [8], and with prior reports of older patients diagnosed only after multiple recurrent strokes [6]. Six of our seven patients had prior cerebrovascular events or chronic infarcts, and several recurred despite antiplatelet or anticoagulant therapy, the clinical pattern most likely to prompt a hypercoagulable evaluation and most likely to yield a positive result.

Distinguishing true APS from incidental aPL positivity

The central interpretive challenge, and the one a reader should hold this series to, is whether the antibodies identified are causally implicated or are incidental. This caution is not theoretical: in the Antiphospholipid Antibodies and Stroke Study (APASS), conducted within the Warfarin-Aspirin Recurrent Stroke Study, baseline aPL positivity neither predicted recurrent thrombo-occlusive events over two years nor altered the response to warfarin versus aspirin, and the investigators concluded that routine aPL screening after ischemic stroke was not warranted [11]. More recent prospective cohort data, however, suggest aPL positivity may still carry prognostic significance for recurrent ischemic stroke in some populations [12], indicating that the clinical relevance of aPL findings remains an evolving, cohort-dependent question rather than one settled by APASS alone. A single positive test, a low-titer result, or an isolated IgM (which can arise transiently with infection, malignancy, or advanced age) carries far less weight than a persistent, high-titer, multi-positive profile. Our cohort spans this entire spectrum. The strongest case is patient #1 (triple positive, markedly elevated titers, persistence confirmed at ≥12 weeks, and recurrence on antiplatelet therapy); patient #3 was also triple-positive but confirmatory testing remains pending. At the other end, patients #5 and #6 had isolated lupus anticoagulant on a single panel, and patients #4 and #7 had IgM-only double positivity on single panels, profiles that score lower and are less specific under the 2023 ACR/EULAR criteria, which were explicitly constructed to raise specificity (99% versus 86% for the 2006 criteria) and which require persistence [2]. Under the 2023 ACR/EULAR criteria, isolated IgM positivity without a qualifying IgG or LA result does not satisfy the laboratory domain [2,13]. Patients #4 and #7 therefore do not meet formal 2023 APS classification and are more accurately characterized as ischemic stroke with aPL antibody positivity of uncertain clinical significance, rather than as classified APS (Table 2). Framing the series as a graded set of attributions rather than seven equivalent 'APS strokes' is both more honest and more useful to the reader, and it is the appropriate response to the APASS critique rather than an argument.

Competing mechanisms and attribution in older patients

Older patients accumulate competing stroke mechanisms, and our cohort is no exception: a PFO or intracardiac shunt was present in patients #1, #4, and #6; large-artery atherosclerosis, carotid stenting, or ulcerated aortic-arch plaque in patients #2 and #7; and severe small-vessel ischemic disease in patient #4. In several patients, the aPL antibodies are therefore best understood as a contributing prothrombotic factor superimposed on conventional vascular disease rather than as the sole cause. This is a meaningful departure from the younger APS patient in whom aPL more often stands alone [7,14], and it argues for individualized rather than reflexive attribution, weighing antibody profile, persistence, infarct pattern, and the strength of alternative explanations together. This pattern is consistent with broader stroke literature showing that thorough etiologic evaluation in older patients disproportionately uncovers complex atherosclerotic and embolic sources compared with younger cohorts [15].

Therapeutic implications

When APS is judged to be operative, the identification carries direct therapeutic weight. Current guidance recommends a vitamin K antagonist (VKA) with a target INR of 2-3 for a first arterial event, with intensified strategies considered for recurrence, and explicitly advises against direct oral anticoagulants in high-risk (triple-positive) disease [16]. Two randomized trials found that rivaroxaban was inferior to a VKA, with an excess of arterial and stroke events [17,18]. This caution is not limited to triple-positive disease: the International Society on Thrombosis and Haemostasis (ISTH) Scientific and Standardization Committee guidance recommends against direct oral anticoagulant use in APS patients with prior arterial thrombosis regardless of antibody profile, reserving direct oral anticoagulants for selected venous-only, non-triple-positive cases [19]. Our cohort reflects this in practice: all seven patients were treated with warfarin, and patient #2 had experienced recurrent strokes during prior anticoagulation that included a direct oral agent. At the same time, the bleeding hazard of anticoagulation in the very elderly is real, as patient #4's supratherapeutic INR and gross hematuria illustrate. The decision to commit an older patient to lifelong VKA therefore depends heavily on diagnostic confidence, which returns the emphasis to persistent, high-specificity confirmation.

Limitations

This is a small, retrospective, single-center series without a comparison group, and it is subject to selection and referral bias toward patients in whom aPL was both tested and found. Because aPL testing was ordered at the discretion of the treating physician rather than through a fixed screening protocol, this series is additionally subject to ascertainment bias, and the true denominator of eligible stroke admissions could not be determined. Antibody profiles were heterogeneous, and persistence at ≥12 weeks, required for formal classification, was documented for only some patients, with confirmatory testing pending or unavailable for others; the diagnosis in those patients should be regarded as provisional. Titers were not uniformly available for all analytes, evaluation for SLE was not completed in every patient, and follow-up was limited and variable. These constraints temper any causal claim and mean the series should be read as hypothesis-generating.

## Conclusions

Antiphospholipid syndrome should not be dismissed as a disease of the young when evaluating ischemic stroke. In this series of older adults, most with cryptogenic, recurrent, or multifocal infarcts, aPL antibodies were identifiable and, in the more convincing cases, plausibly causal, with management consequences that favored anticoagulation over antiplatelet therapy. We suggest considering APS in the differential of embolic stroke of undetermined source and of recurrent stroke in older patients, while insisting on persistent, high-specificity antibody confirmation and careful weighting of competing mechanisms before attributing the event to APS or committing a patient to lifelong anticoagulation. This is hypothesis-generating rather than a call for routine post-stroke aPL screening. We suggest targeted testing in cryptogenic, recurrent, or multifocal infarcts instead.
